# Shexiang Baoxin Pill, Derived From the Traditional Chinese Medicine, Provides Protective Roles Against Cardiovascular Diseases

**DOI:** 10.3389/fphar.2018.01161

**Published:** 2018-11-14

**Authors:** Li Lu, Xiaodong Sun, Chen Chen, Yating Qin, Xiaomei Guo

**Affiliations:** ^1^Department of Cardiology, Tongji Hospital, Tongji Medical College, Huazhong University of Science and Technology, Wuhan, China; ^2^Department of Orthopedics, Puai Hospital, Tongji Medical College, Huazhong University of Science and Technology, Wuhan, China

**Keywords:** Shexiang Baoxin Pill, traditional Chinese medicine, cardiovascular disease, angiogenesis, cardiac remodeling

## Abstract

Shexiang Baoxin Pill (SBP), derived from the traditional Chinese medicine, has been broadly applied for the treatment of cardiovascular diseases including coronary heart disease, heart failure, and hypertension in East Asia for decades. Emerging pharmacological studies have revealed that SBP displays pleiotropic roles in protecting the cardiovascular system, as seen by the promotion of angiogenesis, amelioration of inflammation, improvement of endothelium dysfunction, mitigation of dyslipidemia, repression of vascular smooth muscle cell proliferation, and migration and restraint of cardiac remodeling. In terms of clinical practice, the clinical trials and meta-analyses have proved the efficacy and safety of SBP. In this review, we, for the first time, systematically summarize the cardioprotective effects and underlying mechanisms of SBP and provide novel insights into future research directions of SBP based on the experimental and clinical perspectives.

## Introduction

It is well-recognized that cardiovascular diseases (CVDs) are regarded as the leading cause of death worldwide. Despite the rapid development of western medicines in treating CVDs, the mortality of CVDs still remains high. In developed countries of North America, nearly 170 health losses per 100000 were attributed to CVDs in 2015 (Roth et al., 2017). Moreover, in developing countries of Asia, the mortality of CVDs was 298 cases per 100000 in the same year (Ma et al., 2018). It is imperative to find other remedies for improving the treatment and prognosis of CVDs. Complementary and alternative therapeutics such as traditional Chinese medicines (TCMs) are emerging as crucial protective agents for the cardiovascular system, considering their effective roles in inhibiting pathogenic events of CVDs (Liu and Huang, 2016; Hao et al., 2017).

**FIGURE 1:**
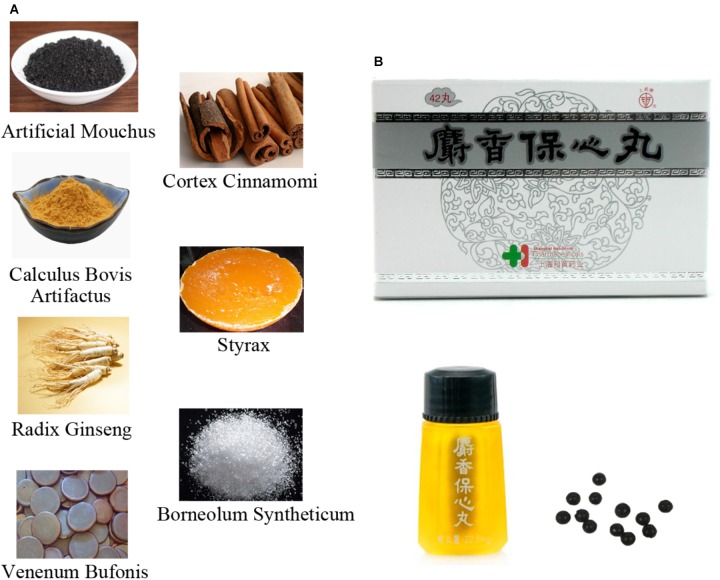
The components included in Shexiang Baoxin Pills and the related Chinese patent medicine. **(A)** The morphology of seven raw medicinal materials which compose Shexiang Baoxin Pills. **(B)** The relevant patent medicine of Shexiang Baoxin Pills manufactured by Shanghai Hutchison Pharmaceuticals, which have been used in clinical practice for several years. The picture of this patent medicine has been permitted to be presented in the manuscript by Shanghai Hutchison Pharmaceuticals.

Shexiang Baoxin Pill (SBP), a classic patent medicine derived from the TCM formula Suhexiang Pill of Song Dynasty in China, has been extensively used for the prevention and treatment of CVDs, such as unstable angina, myocardial infarction (MI), and heart failure (HF) (Zhou et al., 2016; Dong T. et al., 2018). There are seven TCM materials contained in the SBP: Artificial Moschus, Radix Ginseng (Root of Panax ginseng C.A.Mey.), Calculus Bovis Artifactus, Cortex Cinnamomi (Bark of *Cinnamomum cassia* Presl), Styrax (balsam of Liquidambar orientalis Mill.), Venenum Bufonis, and Borneolum Syntheticum (Resin of *Dryobalanops aromatica* C.F.Gaertn) (Figure 1). From the perspective of preclinical studies, SBP has demonstrated therapeutic effects on CVDs via various beneficial mechanisms including regulating angiogenesis and coronary artery dilation, repressing inflammation and oxidation stress, improving lipid metabolism, and protecting vascular endothelium (Ning et al., 2011; Xu et al., 2011; Zhang et al., 2017; Wei et al., 2018). Additionally, in terms of clinical practice, the cardiovascular protective roles of SBP have been proven by several randomized controlled trials and expert consensuses on SBP treatment of CVDs (Zhou et al., 2016; Dong T. et al., 2018; Fan et al., 2018). In the following sections, we provide an overview of basic experiments and clinical studies on the cardiovascular effects of SBP and the underlying mechanism profiles.

## Pharmacological Features of SBP

### Active Components

Using chromatography and mass spectrometry techniques, more than 70 non-volatile and 40 volatile compounds in SBP have been identified by *in vitro* studies, including cholic acid, deoxycholic acid, cinobufagin, and ginsenoside Rb1 (Liu et al., 2015; Lv et al., 2017). The internal related substances after oral administration of SBP have also been widely analyzed during the past years. Jiang et al. (2009) found that 17 compounds and 4 metabolites were present in the plasma of rats after SBP uptake, which contained gamabufotalin, resibufaginol, ginsenoside Re, chenodeoxycholic acid, and 17-hydroxyprogesterone. Using a sensitive mass spectrometry method, another study determined 4 volatile components in rat plasma after gastric perfusion with SBP, including isoborneol, borneol, muscone, and cinnamaldehyde (Chang et al., 2014). Moreover, as the main constituents with therapeutic roles and cardiotoxic effects in SBP separately, ginsenosides from Radix Ginseng and bufadienolides from Venenum Bufonis were rapidly absorbed into the blood, and the pharmacokinetic characteristics of their metabolites have been investigated for improving the efficacy and safety of drug application (Table 1; Huang et al., 2015; Peng et al., 2015; Tao et al., 2017).

**Table 1:** The main chemical components included in the raw medicinal materials of SBP.

Medicinal materials	Bioactive components	Reference
Artificial Moschus	Muscone, testosterone	Fang et al., 2017
Radix Ginseng	Ginsenoside Ra1/2, Rb1/2/3, Rc, Rd, Re, Rf, and Rg1/2/3	Tao et al., 2017
Calculus Bovis Artifactus	Cholic acid, deoxycholic acid, ursodeoxycholic acid, chenodeoxycholic acid, hyodeoxycholic acid, bilirubin, and cholesterol	Jiang et al., 2009
Cortex Cinnamomi	Cinnamaldehyde and cinnamic acid	Lv et al., 2017
Styrax	Benzyl benzoate	Liu et al., 2015
Venenum Bufonis	Cinobufagin, resibufogenin, resibufagin, gamabufotalin, bufalin, 1β-hydroxylbufalin, arenobufagin, bufotalin, telocinobufagin, and telibufagin	Tao et al., 2017
Borneolum Syntheticum	Borneol and isoborneol	Chang et al., 2014


### Herb-Drug Interaction

Shexiang Baoxin Pill has been prescribed alone or in combination with other drugs for the treatment of CVDs over the years (Chen et al., 2018; Fan et al., 2018). Considering that SBP affects the activities and levels of several enzymes which regulate the metabolisms of some clinical drugs, combination therapies combining SBP with modern drugs have gained increasing attention for reducing the potential of adverse effects (Shen et al., 2016). For instance, cytochrome P450s (CYP450s) represent a group of hemeproteins catalyzing biotransformation of the majority of medications and SBP has shown to modulate the activities and expressions of catalytic enzymes among CYP450s, thereby suggesting that SBP might affect pharmacokinetic profiles of drugs metabolized by CYP450s (Jiang et al., 2012; Shen et al., 2016). According to a study reported by Tao et al., increased C_max_ and AUC_(0-t)_ and reduced T_1/2_ and T_max_ of simvastatin were seen in plasma after simvastatin was co-administrated with SBP (Tao et al., 2016). Thus, changes in drug metabolism parameters induced by the combination therapies and related mechanisms should be further explored to guide clinical drug applications.

## Protective Mechanisms of SBP

### Promotion of Angiogenesis

Angiogenesis is a pivotal protective action in the ischemia pathologic environment, which induces the formation of new capillaries and ensures blood and oxygen supply that accelerates damage restoration (Sun et al., 2018). Previous studies have established that SBP is capable of alleviating arterial obstructive disorders via facilitating angiogenesis activities (Zhang et al., 2017; Guo et al., 2018). Huang F. et al. (2017) found that SBP significantly reduced the infarction area and enhanced cardiac functions of Sprague-Dawley rats with MI. They demonstrated that this cardioprotective role was attributed to SBP-induced angiogenesis in pert-infarct areas, and the underlying mechanism was associated with SBP-mediated level increase of serum 20-hydroxyeicosatetraenoic acid, which upregulated vascular endothelial growth factor (VEGF) expression and then boosted circulating endothelial progenitor cells (EPCs) to proliferate and accumulate to the injured region for participating in angiogenic events (Huang F. et al., 2017). In addition, under the stimulation of sheer stress, SBP enabled the EPCs to possess increased abilities of angiogenesis by means of inducing endothelial nitric oxide synthase (eNOS) expression and subsequent generation of nitric oxide (NO) that was considered as an agonist of vessel formation (Li G. et al., 2015). Moreover, cinnamaldehyde, a bioactive ingredient from SBP, has been proven to exert pro-angiogenic properties by heightening the proliferation, mobilization, and tube formation of endothelial cells (ECs) through stimulating specific molecules and pathways responsible for pro-angiogenic processes such as VEGF, PI3K/Akt, and MAPK signaling cascade (Yuan et al., 2018). Other research indicated that SBP and its component ginsenosides shared similar pharmacological effects as cinnamaldehyde on ECs, whereas the possible mechanisms of the results remained unknown, probably linked to angiogenic modulators including LATS1/2/YAP/CTGF and VEGFR1 cascade which were targeted by SBP (Lv et al., 2014; Fang et al., 2017; Wu et al., 2018). In an animal experiment regarding MI, SBP decreased the MI area and increase microvessel count via improving angiogenesis, as evidenced by elevation of VEGF and basic fibroblastic growth factor (Wang et al., 2002). On an interesting note, some scholars declare that SBP repress angiogenic processes in the atherosclerotic lesions for enhancing the stabilization of plaques (Li et al., 2006; Shen et al., 2010). This phenomenon indicates that the effects produced by SBP on angiogenesis might vary among different pathophysiological environments, and illuminating the precise mechanisms is valuable for providing new insights into the protective roles of SBP against CVDs.

### Amelioration of Inflammation

A substantial number of investigations have demonstrated that inflammation plays a vital role in the initiation and progression of CVDs. Inflammatory cells and their secreted cytokines destroy the integrity of vascular endothelium, which result in LDL-C disposition and oxidation in the subendothelium and the transendothelial recruitment and foam cell transformation of macrophages, favoring the development of atherosclerosis (AS). In addition, the inflammatory state increases the consumption of energy and oxygen, which adds to the burden of the heart pumping blood, and leads to raised mortality of MI and chronic HF (Siti et al., 2015; Shapiro and Fazio, 2016). There is also evidence that inflammation is able to trigger cardiac arrhythmia by promoting electrical and structural remodeling (Hu et al., 2015). Then, inhibiting inflammation is a promising approach for treating CVDs Xu et al. (2011) elaborated that pretreatment with SBP, in a dose-dependent manner, reversed the increment of MCP-1 and IL-6 in ECs induced by H_2_O_2_ by lowering the content of inflammatory transcriptional factor NF-κB. In terms of anti-AS, Tao et al. observed that SBP alleviated the atherogenesis in rabbits with a high fat diet, accompanied by decrement of TNF-α, IL-6, and IL-8, which was partially related with its anti-inflammatory activity by blocking Ca^2+^/CaMKII/TLR/NF-κB axis (Tao et al., 2015). With network pharmacology-based methods, several inflammation related molecules were identified to be targeted by SBP, including ICAM-1, COX-2, TLR4, and VCAM-1 (Fang et al., 2017). Mounting evidence in literature increasing evidence in the published papers have suggested that SBP is capable of mitigating the progression of hypertensive nephropathy and cardiotoxicity by diminishing the tissue inflammatory responses, as seen by the decline in TNF-α, iNOS, TGF-β1, IL-1β, and ICAM-1 (Hu et al., 2009; Tian et al., 2011). In addition, damage of cardiac cells triggered by high glucose was ameliorated when cells co-incubated with SBP, which was due to the suppression of p38 MAPK and NF-κB pathway, thereby indicating the potentials of SBP in diabetic cardiomyopathy treatment (Yang et al., 2016). Studies analyzing serum metabolomics reported that the efficacy of SBP in MI therapy was partly ascribed to the anti-inflammatory abilities of SBP, given that the detected biomarkers involved in inflammation such as PGE2 and 12(S)-HETE were down-regulated by SBP (Xiang et al., 2012, 2013). Moreover, SBP was found to depress the incidence of arrhythmias and improved ventricular remodeling in rats with MI by decreasing the expression of inflammation cytokines including IL-18 and TNF-α in the damaged areas (Xu et al., 2015; Yang B. et al., 2015). Metabolic syndrome (MS), characterized by dyslipidemia, hypertension, and hyperglycemia, is thought to be a key contributor to CVDs. It was revealed that SBP provided the cardioprotective actions by abrogating the development of MS, impeding NF-κB, TNF-α, and IL-6 expression and raising the level of IL-10 and PPAR-γ, an anti-inflammatory factor encumbering the activation of NF-κB (Li Y. et al., 2015; Wei et al., 2018). Therefore, SBP exerts anti-inflammatory roles, mainly by decreasing the expression and activation of NF-κB, thereby suggesting the potential contribution of NF-κB suppression to the cardioprotective effects of SBP.

### Improvement of Endothelium Dysfunction

Recovering and maintaining the normal function of endothelium has been proposed as a promising approach for treating CVDs, since dysfunction of endothelium is a pivotal pathogenic event of disease development of the cardiovascular system. When exposed to diverse stimuli, the endothelium barrier is damaged and its permeability is increased, which accelerates the accumulation of lipids and leukocytes into the tunica intima, thereby facilitating vascular stenosis followed by the occurrence of ischemic heart disease and stroke (Gimbrone and Garcia-Cardena, 2016). As endothelium dysfunction leads to the reduction of some endothelial-dependent vasoactive substances such as NO, the vasodilation property is impaired and related vascular stiffness is raised, thereby elevating the blood pressure and aggravating hypertension (Oparil et al., 2018). Reactive oxygen species (ROS) and oxidative stress are known as the principal pathological elements implicated in endothelial injury (Ungvari et al., 2018). Emerging evidences have clarified that SBP could diminish H_2_O_2_-provoked ECs apoptosis and improve cell proliferative activity. The underlying mechanism involves anti-oxidative effects of SBP-induced level increase of SOD and expression reduction of MDA and NADPH oxidase p22phox subunit (Ning et al., 2011). Additionally, SBP was reported to enhance the production of NO via boosting eNOS activity, which might be another mechanism by which SBP protected ECs from ROS-induced damage, considering the fact that NO played key roles in regulating anti-apoptotic and anti-oxidative abilities of ECs (Li et al., 1999; Ungvari et al., 2018). In an experiment model of cerebral ischemia reperfusion injury, SBP was found to elevate the Bcl-2 level and lower the caspase-3 level for protecting the brain cells, but the relevant apoptotic pathways modulated by SBP involved in improvement of endothelium dysfunction are still required to be elucidated (Chen et al., 2008). Furthermore, SBP has been revealed to exhibit the therapeutic endothelial protection in patients with CVDs by raising NO and SOD and reducing ET-1 and MDA (Zhang Y. et al., 2016).

### Mitigation of Dyslipidemia

Both the basic research and clinical trials have documented that disorder of lipid metabolism is deeply associated with progression of AS and its sequelae (Shapiro and Fazio, 2016). Agents lowering lipids in the circulation display great implications in preventing and treating atherogenesis (Hurtubise et al., 2016). Yu et al. discovered that SBP lessened the contents of blood TG, TC, and LDL-C, while augmenting the level of HDL-C in rabbits that received high fat diets accompanied by the decline in thickness of plaques and intima-media thickness in the abdominal aorta, which may possibly be linked to SBP-evoked decreased lipid sedimentation in the vascular wall (Yu et al., 2012a). Furthermore, the mechanism underlying SBP modulating dyslipidemia has also been investigated. There was evidence showing that SBP was able to elevate the expression of key molecules required for the pathways of fatty acid oxidation, including AMPK, PPAR-α, and PGC-1α, resulting in catabolism and decline of TG (Li Y. et al., 2015; Wei et al., 2018). As PPAR-γ signaling is responsible for HDL-C biosynthesis in the liver and release for circulation, SBP has been shown to promote PPAR-γ expression in cardiac tissues, and the influence of SBP on PPAR-γ level in hepatocytes should be analyzed (Li Y. et al., 2015). In addition, histological examinations revealed that SBP significantly mitigated AS development and weakened the expression of LOX-1 in atheroma lesions (Zhu et al., 2008). As LOX-1 belongs to the group of scavenger receptors which promote macrophages to uptake ox-LDL and switch into foam cells, blockade of foam cell formation might be another important mechanism of SBP for delaying AS progression. As LOX-1 belongs to the group of scavenger receptors promoting macrophages uptake of ox-LDL and switch into foam cells, these findings suggest that a blockade of foam cell formation is another important mechanism of SBP for delaying AS progression.

### Proliferation and Migration Repression of Vascular Smooth Muscle Cells

Uncontrolled proliferation and migration of vascular smooth muscle cells (VSMCs) is a hallmark of CVDs such as AS, restenosis after percutaneous coronary intervention (PCI), and hypertension. Under normal situations, VSMCs keep a resting status termed as contractile phenotype in the tunica media, but they change into the unchecked synthetic phenotype and then proliferate and mobilize into the subendothelial layer, thereby leading to intimal thickening, in response to diverse stimuli such as inflammatory cytokines and stress damage. After switching into the synthetic state, VSMCs possess lessened levels of differentiation biomarkers including α-SMA, SM22α, and SM-MHC, except for exhibiting enhanced growth and migration capabilities (Bennett et al., 2016; Durgin and Straub, 2018; Liu et al., 2018). Che et al. (2010) reported that the expression of α-SMA and SM-MHC in VSMCs was elevated after co-administration of VSMCs with different concentrations of SBP. Similarly, Zhang et al. (2014) showed that SBP increased the positive rate of VSMCs expressing α-SMA and SM-MHC while arresting the cell cycle at G1 phase. These findings hint that SBP might perform anti-proliferative functions on VMSCs by expediting the phenotypic switch from the synthetic to contractile state. With respect to the effects of SBP on VSMCs mobilization, another study revealed that SBP could prevent restenosis following stenting by impeding neointimal formation by repressing the movement of VSMCs (Yu et al., 2012b).

### Restraint of Cardiac Remodeling

In view of the limited regenerative capacity of mature cardiomyocytes, loss of functional myocardial cells induces the remaining myocardium to endeavor and compensate for the decrease of contractile function and generate excessive extracellular matrix, leading to myocardial hypertrophy, structure disorder and fibrosis, termed as cardiac remodeling, a common pathological change in HF post-MI, hypertensive and diabetic cardiomyopathy (Dong Y. et al., 2018). A study monitoring the roles of SBP in the cardiac pathological changes of diabetic rats showed that SBP significantly alleviated myocardial interstitial fibrosis, reversed disorder of fiber arrangement and mitochondrial swelling, and improved the systolic and diastolic functions, which was probably due to the depressed levels of TGF-β1 and Ang II in local tissues (Liu et al., 2012). Xu and Zhang (2016) also discovered that SBP could prohibit the activation of TGF-β1/Smads pathway to encumber the development of hypertension-provoked myocardium fibrosis. In addition, according to Jiang et al. (2011), a few down-regulated biomarkers comprising of corticosterone, aldosterone, and cortisol were detected in the MI rats’ serum treated with SBP. Since these affected substances were metabolites of steroid hormone biosynthesis and facilitated the occurrence of hypertrophy, interruption of the pathway of steroid hormone generation appeared to be responsible for the suppressive effects of SBP on cardiac hypertrophy (Jiang et al., 2011). Moreover, previous evidence suggests that restoring the balance between MMP-9/TIMP-1 was a critical mechanism underlying SBP-impeded cardiac interstitium remodeling in rabbits with MI-evoked HF (Huang et al., 2011). Considering that aberrant activation of nerval and humoral factors had been indicated to perform positive effects on HF progression, gavaging with SBP increased the left ventricular ejection fraction (LVEF) and improved cardiac functions of rats suffering from HF. The mechanism was likely to be attributed to signal transduction modulation of the sympathetic and renin-angiotensin system, as seen by SBP-induced level alterations of α_1_ an β adrenergic receptors and angiotensin II (Ang II) type 1 and type 2 receptors (Cao et al., 2011; Li et al., 2012).

Furthermore, pharmacological experiments have depicted that SBP could attenuate the dysfunction of energy metabolism aroused in MI animal models while reducing the size of necrotic and fibrotic tissues (Fan et al., 2011; Xiang et al., 2012, 2013). Because of the vital roles of energy metabolism in keeping the organs functioning adequately, SBP has the potential to prevent the aggravation of cardiac remodeling by mediating the energy metabolism pathways.

## SBP Treatment for CVDs

### Unstable Angina

It is well-known that acute coronary syndrome (ACS) can be divided into two categories: unstable angina and MI. Clinical observations and retrospective analyses have clarified the effectiveness of SBP in treating unstable angina pectoris. Xing et al. reported that SBP had more advantages in depressing the frequency of an angina attack than isosorbide mononitrate tablets in patients with unstable angina (Xing et al., 2015). Yang and Zhang (2016) observed the safety and efficacy of SBP in elderly patients. They showed that SBP reversed the abnormal manifestation of electrocardiogram (ECG), improved the hemodynamics and coagulation risk, and enhanced the vasodilatation ability without producing obvious adverse effects, thereby likely lowering the incidence of acute thrombotic events. Several scholars implemented the trials involving combination drug therapy and found that the therapeutic profiles of the combination strategies amalgamating SBP with conventional medications were superior to that of the routine western drugs alone, which was explained by decrease of frequency and duration of angina occurrence, decrement of nitroglycerin consumption, restoration of ECG presentation, and elimination of serum inflammatory and oxidative factors (Zhang, 2013; Xie and Wang, 2016; Jiang et al., 2017).

### Myocardial Infarction

In the cases of MI, a lot of evidence has demonstrated that SBP exhibits high efficiency in disease therapy. For example, studies compared SBP combined with conventional remedies versus conventional therapies alone for management of ST-segment elevation MI after PCI. These studies showed that SBP remarkably improved LVEF and NT-proBNP level, diminished the contents of myocardial injury markers including CK, cTNT, and cTNI, and decreased the overall rate of adverse cardiovascular events (Liu, 2016; Zhang and Guan, 2016). In a meta-analysis evaluating the effects of SBP in treating non-ST-segment elevation MI, decline in risks of cardiovascular events and hospitalizations and remission of angina symptoms and abnormal ECG findings with good tolerance were observed in patients after long-term oral administration of SBP (Zhou et al., 2016). Moreover, according to Li et al. (2018), SBP proved to enhance the blood flow of stented artery and related myocardial perfusion following stent implantation, thereby hinting the remedial hope of SBP in slow-flow/no-flow post-PCI, which was possibly linked to protective roles of SBP against endothelium dysfunction. It was demonstrated that severe inflammatory reactions occured during the MI episode, which could induce the lethal arrhythmia (Hu et al., 2015; Siti et al., 2015). There was evidence that SBP improved QT interval dispersion and the heart rate variability, decelerating the incidence of ventricular arrhythmia in patients experiencing MI (Luo, 2015; Feng et al., 2017). Together with the fact that SBP serves as an anti-inflammatory agent, SBP appears to encumber the occurrence of MI-related arrhythmia via abrogating inflammatory responses, while the specific mechanisms warrant further exploration. Additionally, Zhang L. et al. (2016) found that SBP was able to mitigate clopidogrel resistance in ACS participants by improving the platelet aggregation rate. With regard to thrombolytic therapy, Chen and Ma (2013) and Yang F. et al. (2015) drew a conclusion that SBP, when added to the routine thrombolytic program, produced better therapeutic influences on MI patients, as indicated by decrement of blood LDL and TG, enhancement of cardiac function, attenuation of myocardial impairment, and protection of vascular endothelium.

### Heart Failure

As the advanced stage of multiple CVDs, HF with high death rates pose a great threat to quality of life, it also places a heavy burden on the whole of society (Roth et al., 2017). SBP has been broadly applied as an adjuvant treatment in HF and the corresponding pharmacological effects have been extensively investigated in recent years. Ding et al. assessed the clinical efficacy of SBP on the treatment of chronic ischemic HF. Following up after 6 months, the authors discovered that SBP substantially elevated the level of LVEF and 6-min walk distance (6-MWD) and depressed the value of BNP (Ding et al., 2013). In the cases of coronary heart disease (CHD)-related HF, a combination of SBP and conventional drugs exerted preferable effects compared to conventional medications, shown by the normalization of indicators reflecting cardiac function including LVEF, left ventricular end diastolic and systolic dimension, and cardiac output (CO), and decrease of specific HF biomarker NT-proBNP (Li R. et al., 2015; Huang, 2017). Additionally, the pooled data of a meta-analysis completed by Dong T. et al. (2018) mentioned that SBP was demonstrated to be effective and safe in suppressing HF progression, on account of the increment of LVEF, CO, stroke volume, and 6-MWD and decrement of BNP and NT pro-BNP without apparent side effects. In regard to HF with normal ejection fraction, SBP along with conventional treatment showed a more favorable impact than the conventional strategy on enhancing exercise tolerance, cardiopulmonary function, and life quality of patients (Huang M. et al., 2017). Kong et al. (2015) observed the therapeutic effectiveness of SBP on acute HF post-MI and reported that BNP levels and rates of all-cause death and cardiac death were lowered and the LVEF value was lifted after 2 months of SBP administration. Moreover, emerging evidence has established that some chemotherapy drugs exhibit cardiotoxicity and could impair the cardiac function, causing patients to suffer from HF (Moslehi et al., 2017). Recently, it was shown that SBP alleviated damages of myocardium and cardiac function triggered by doxorubicin via unknown mechanisms, as seen by improved levels of cTNI, CK-MB, BNP, LVEF, and fractional shortening (Qi et al., 2017). Thus, given the above studies, SBP is worthy of clinical application for mitigating HF development and improving the prognosis of patients.

### Hypertension

According to the latest epidemiological data, approximately one billion of the global population are diagnosed with arterial hypertension, with nearly an estimated 270 million affected cohort in China (Forouzanfar et al., 2017; Ma et al., 2018). SBP has been used as complementary agent for the treatment of hypertension and relevant complications in Chinese patients for decades. Huang (2009) indicated that SBP added to amlodipine produced clinical efficacy superior to amlodipine alone in depressing the systolic pressure and pulse pressure of patients with isolated systolic hypertension. Trials concerning the management of hypertension with left ventricular hypertrophy reported that adjunctive therapy of SBP greatly lowered blood pressure, relieved clinical symptoms such as palpitations, chest stuffiness, and anhelation, and improved left ventricular posterior wall thickness, left ventricular mass index, interventricular septal thickness, and ECG parameters (Chen et al., 2012; Zhang Y. et al., 2016). In addition, SBP along with routine medications displayed higher efficiency in anti-hypertensive and cardiotonic roles in cases of hypertension combined with HF (Liu et al., 2017).

### Safety and Adverse Effects

Despite the beneficial roles of SBP in the treatment of CVDs, the clinical safety and potential adverse reactions of SBP cannot be ignored. Previous toxicologic studies showed that TG levels and coagulation parameters were changed within a short time in very few female rats after oral administration with SBP at nearly 90 multiples of the human dosage, which implies that it might be advisable to monitor coagulation indexes and blood lipids of female patients. The side effects of SBP mainly include tongue numbness, nausea, emesis, rashes, dizziness, and palpitations. Several clinical trials have confirmed that the incidence rate of adverse effects is less than 0.01% and patients tolerate these side effects well. In addition, considering the potential cardiotoxicity of Venenum Bufonis in SBP, there is evidence indicating that other medicinal materials in SBP markedly alleviate the toxicity of Venenum Bufonis, thereby facilitating the safety of SBP in clinical practice (Wei et al., 2015).

## Conclusion

**FIGURE 2:**
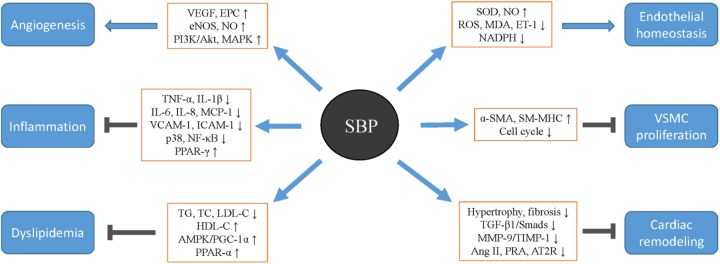
The therapeutic roles by which Shexiang Baoxin Pills protect against cardiovascular diseases. SBP, Shexiang Baoxin Pill; VEGF, vascular endothelial growth factor; EPC, endothelial progenitor cell; eNOS, endothelial nitric oxide synthase; NO, nitric oxide; PPAR, peroxisome proliferator-activated receptor; AMPK, AMP-activated protein kinase; PGC-1α, peroxisome proliferator-activated receptor gamma coactivator 1-α; SOD, superoxide dismutase; MDA, malondialdehyde; ET-1, endothelin-1; α-SMA, α-smooth muscle actin; SM-MHC, smooth muscle myosin heavy chain; MMP-9, matrix metalloprotein 9; TIMP-1, tissue inhibitor of metalloproteinase 1; PRA, plasma rein activity; AT2R, angiotensin II type 2 receptor.

TCMs are widely associated with disease prevention and treatment (Liu and Huang, 2016). SBP, derived from the ancient TCM formula, has demonstrated to directly exert beneficial therapeutic effects on CVDs including CHD, HF, and hypertension (Table 2), by means of promoting angiogenesis, inhibiting inflammation, improving endothelial function, and dyslipidemia, as well as interfering with VSMCs growth and cardiac remolding (Figure 2). In light of the pharmacological and therapeutic profiles in the cardiovascular system discussed above, SBP is deserving of further clinical promotion and application. At the same time, more experimental and clinical tests need to be conducted in the near future to provide more reliable evidence supporting SBP use. SBP is a mixture composed of seven raw medicinal materials and research currently mainly focuses on the pharmacokinetics of compounds in Radix Ginseng and Venenum Bufonis. The pharmacokinetic features of other ingredients such as the components in Artificial Moschus and Calculus Bovis Artifactus are scarce and should be analyzed to determine the effectiveness and safety of SBP. Moreover, to date, most basic studies investigating the protective mechanisms of SBP against CVDs detect the influences of SBP on the pathogenic phenomena and downstream effector molecules, while not exploring the key upstream signal pathways by which SBP affect disease development, leading to the ambiguity of functional signaling networks of SBP. It is therefore necessary for continual studies on the specific pathways involved in the cardioprotective mechanisms of SBP to lay a credible theoretical foundation. In addition, clinical trials observing the therapeutic efficacy of SBP on CVDs are characterized by small sample sizes, low methodologic quality, ill-defined bias risk, and short-term follow-ups, which to some extent decreases the credibility of the clinical data. Therefore, randomized, controlled, and double-blind trials with large sample sizes and long-term follow-ups are urgently needed to affirm efficacy and prospect of SBP in CVDs management.

**Table 2:** The therapeutic effects of SBP on the management of CVDs.

Clinical application	Therapeutic effects	Reference
Unstable angina	Angina symptom, nitroglycerin consumption ↓	Xing et al., 2015
	Abnormal ECG parameters, hemodynamics, coagulation risks ↓	Yang et al., 2016
	Hs-CRP, MMP-9, MDA, hcy, ET-1 ↓	Xie and Wang, 2016; Zhang, 2013
	Vasodilatation, SOD and NO ↑	Zhang, 2013; Yang et al., 2016
Myocardial infarction	CK, CK-MB, LDH, BNP, NT-proBNP, cTNI, cTNT, H-FABP ↓	Liu, 2016; Zhang and Guan, 2016
	MACE risks, hospitalization, angina symptom, ECG abnormity ↓	Zhou et al., 2016
	QTd, QTcd, Platelet aggregation rate ↓	Feng et al., 2017
	MMP-2, NLR, suPAR, IL-6, hs-CRP, LDL, TG, vWF ↓	Chen and Ma, 2013; Li et al., 2018
	LVEF, TIMI flow, SOD, FMD ↑	Yang F. et al., 2015; Li et al., 2018
	PNN50, RMSSD, SDNN, SDANN ↑	Feng et al., 2017
Heart failure	BNP, ET-1, NT-proBNP, cTNI, CK-MB, H-FABP ↓	Ding et al., 2013; Qi et al., 2017
	LVEDD, LVESD, FS, A/E ↓	Li R. et al., 2015; Qi et al., 2017
	Dyspnea, all-cause death, cardiac death ↓	Kong et al., 2015
	6-MWD, LVEF, NO, CO, SV ↑	Li R. et al., 2015; Huang, 2017
Hypertension	SBP, DBP, pulse pressure ↓	Huang, 2009; Chen et al., 2012
	LVPWT, LVDD, IVST, LVMI ↓	Zhang Y. et al., 2016
	FMD, EID, MDA, LPO, ET-1 ↓	Zhang Y. et al., 2016
	Clinical manifestations ↓	Chen et al., 2012
	SOD, TAC, NO ↑	Zhang Y. et al., 2016


*Hs-CRP, hypersensitive C-reactive protein; hcy, homocysteine; CK, creatine kinase; CK-MB, creatine kinase isoenzymes; LDH, lactate dehydrogenase; BNP, brain natriuretic peptide; NT-proBNP, N-terminal pro-brain natriuretic peptide; cTNI, cardiac troponin I; cTNT, cardiac troponin T; H-FABP, heart fatty acid-binding protein; MACE, major adverse cardiovascular events; QTcd, heart-rate corrected QT interval dispersion; NLR, neutrophil to lymphocyte ratio; TIMI, thrombolysis in myocardial infarction; FMD, flow-mediated dilatation; PNN50, percentage of adjacent NN intervals differing by more than 50 milliseconds; RMSSD, square root of the mean squared difference of successive RR intervals; SDNN, standard deviation of RR intervals; FS, fractional shortening; A/E, early and late diastolic filling velocity ratio; CO, cardiac output; SV, stroke volume; LVPWT, left ventricular posterior wall thickness; LVDD, left ventricular end diastolic dimension; IVST, interventricular septal thickness; LVMI, left ventricular mass index; EID, endothelium-independent dilatation; TAC, total antioxidative capability.*

## Author Contributions

LL and XG designed this study and wrote the manuscript. XS prepared the figures and tables. CC and YQ compiled relevant research and studies.

## Conflict of Interest Statement

The authors declare that the research was conducted in the absence of any commercial or financial relationships that could be construed as a potential conflict of interest.
